# Melatonin alleviates renal injury by activating mitophagy in diabetic nephropathy

**DOI:** 10.3389/fendo.2022.889729

**Published:** 2022-08-05

**Authors:** Hanfen Tang, Ming Yang, Yinghong Liu, Xuejing Zhu, Shiping Liu, Hong Liu, Lin Sun, Panai Song

**Affiliations:** ^1^ Department of Nephrology, Second Xiangya Hospital, Central South University, Changsha, China; ^2^ Department of Nutrition, Second Xiangya Hospital, Central South University, Changsha, China; ^3^ Key Laboratory of Kidney Disease & Blood Purification in Hunan Province, Institute of Nephrology, Central South University, Changsha, China

**Keywords:** melatonin, mitophagy, diabetic nephropathy (DN), p-AMPK, renal tubular injury

## Abstract

Diabetic nephropathy (DN) causes serious renal tubule and interstitial damage, but effective prevention and treatment measures are lacking. Abnormal mitophagy may be involved in the progression of DN, but its upstream and downstream regulatory mechanisms remain unclear. Melatonin, a pineal hormone associated with circadian rhythms, is involved in regulating mitochondrial homeostasis. Here, we demonstrated abnormal mitophagy in the kidneys of DN mice or high glucose (HG)-treated HK-2 cells, which was accompanied by increased oxidative stress and inflammation. At the same time, the melatonin treatment alleviated kidney damage. After mitochondrial isolation, we found that melatonin promoted AMPK phosphorylation and accelerated the translocation of PINK1 and Parkin to the mitochondria, thereby activating mitophagy, reducing oxidative stress, and inhibiting inflammation. Interestingly, the renal protective effect of melatonin can be partially blocked by downregulation of PINK1 and inhibition of AMPK. Our studies demonstrated for the first time that melatonin plays a protective role in DN through the AMPK-PINK1-mitophagy pathway.

## Introduction

With the development of the social economy and the improvement of living standards, the incidence of diabetes is increasing. Long-term hyperglycemia can lead to a series of microvascular complications in diabetic patients, such as diabetic nephropathy (DN) (1) and diabetic retinopathy (DR) (2). Approximately 30 to 40% of diabetes mellitus (DM) will develop into DN (3). At present, DN has become an important cause of end-stage renal disease (ESRD). However, the prevention and treatment of DN in clinical practice is very limited, and there is a lack of specific drugs to treat it. Therefore, a thorough understanding of its pathogenesis is conducive to developing new medicines for DN. Recently, multiple factors have been reported to be involved in the progression of renal injury in DN, such as hypoxia (4, 5) and oxidative stress (6). However, these factors cannot fully reveal the occurrence and progression of DN. Therefore, it is necessary to further explore the molecular mechanism of DN.

As one of the organs with high metabolism, the kidney contains a large number of mitochondria to maintain its physiological function (7). In the state of DN, the kidney needs a large amount of ATP to resist the influence of high glucose (7), and the mitochondria are in a long-term overloaded state. Damaged mitochondria can release many mitochondrial contents, leading to inflammation and apoptosis (7–10). Therefore, timely clearance of damaged mitochondria can help prevent further damage, a process called mitophagy (11). Some studies have revealed the existence of abnormal mitophagy in the kidneys of DN (12) and activation of mitophagy can alleviate tubular injury in DN (13, 14). However, the upstream and downstream regulatory mechanisms of DN have not been clearly described. Melatonin, a pineal hormone associated with circadian rhythms, is involved in mitochondrial homeostasis regulation (15), energy metabolism (16), lipid regulation (17), and reproduction (18). Interestingly, mitochondrial dysfunction (12), abnormalities of energy homeostasis (19) and disorders of lipid metabolism (20) are closely related to the occurrence of DN. Unfortunately, the role of melatonin in DN has been poorly studied.

This study noted abnormal mitophagy in the kidneys of DN mice or high glucose (HG)-treated HK-2 cells, accompanied by increased oxidative stress and inflammation. Melatonin can phosphorylate AMPK, increase mitophagy and alleviate renal injury, while downregulation of PINK1 or inhibition of AMPK can partially block these effects. Our results suggest that melatonin plays a renoprotective role in DN through the AMPK-PINK1-mitophagy pathway.

## Materials and methods

### Animal models

Eight-week-old C57BL/6 male mice were obtained from Slyke Jingda Biotechnology Company (Hunan, China). All animal models were divided into three groups: the wild type group (WT); DN group; and DN + melatonin group (n = 6). Eight-week-old C57BL/6 male mice were fed a high-fat diet for 1 month and then were subjected to an intraperitoneal injection of STZ (Sigma-Aldrich, 50 mg/kg body weight/day) for 5 consecutive days to induce the diabetic mouse model. Three days after the last injection, the blood glucose level was ≥16.6 mmol/L, and the diabetes mouse model was considered successful. The diabetic mice continued feeding for 12 weeks to induce diabetic kidney damage. For the DN + melatonin group, diabetic mice were treated with melatonin (0.2mg/kg/day) for 12 weeks. The mice were euthanized at 24 weeks, and blood, urine, and kidney samples were collected for subsequent analysis. All animal experiments were approved by the Institutional Committee for Care and Use of Laboratory Animals at Central South University, China.

### Renal histology

Hematoxylin and eosin (HE) and Masson staining of paraffin sections of kidney tissue were performed to observe pathological injury of the kidney. Renal tubulointerstitial injury was evaluated by a semiquantitative scoring system as previously described (21). Briefly, a score of 0 means no interstitial fibrosis and tubular atrophy, while scores of 1, 2 and 3 represent interstitial fibrosis and tubular atrophy areas less than 25 percent, less than 50 percent, and more than 50 percent, respectively.

### Immunohistofluorescence (IHF) staining

Four-micron-thick renal paraffin sections were used for IHF staining. Briefly, after paraffin section dewaxing, rehydration, antigen repair, permeability, and blocking, the renal tissues were incubated with anti-F4/80 rabbit polyclonal antibody (Servicebio, GB11027, 1:500, Wuhan, China), anti-fibronectin (FN) rabbit polyclonal antibody (Servicebio, GB114057, 1:200, Wuhan, China), anti-α-SMA rabbit polyclonal antibody (Servicebio, GB111364, 1:400, Wuhan, China) and anti-NLRP3 rabbit polyclonal antibody (Servicebio, GB11300, 1:600, Wuhan, China) at 4°C overnight. After rewarming, the kidney tissue was incubated with a secondary antibody at room temperature for 1 hour. After staining the nucleus, the renal paraffin sections were observed and photographed under a fluorescence microscope. For costaining, after dewaxing, rehydration, antigen repair, permeability and blocking, anti-LC3B (Proteintech, 14600-1-AP, 1:200) antibody and anti-COXIV antibody (Abcam, ab33985, 1:500) were incubated in renal tissue simultaneously at 4°C overnight. After rewarming, the kidney tissue was incubated with anti-mouse and anti-rabbit secondary antibodies at room temperature for 1 hour simultaneously. Then the nuclei were stained and photographed.

### Dihydroethidium (DHE) staining

6-μm-thick unfixed cryostat sections of renal tissues were stained with the cell-permeable agent dihydroethidium (1 μM, DHE, Sigma-Aldrich) in the dark for 20 min, followed by fluorescence microscopy to assess ROS production in renal tissue.

### Western blotting

The protein concentrations extracted from renal tissue or HK-2 cells were quantified by a BCA Protein Assay Kit (Beyotime Biotechnology, China). Then the samples were mixed with 5× SDS loading buffer and heated it at 95°C for 5 minutes. Equal amounts of proteins were used for western blot analysis.

### Cell culture

The renal human proximal tubular epithelial cell line HK-2 was obtained from ATCC, and DMEM/F12 (Gibco, USA) containing 10% fetal bovine serum (Gibco, USA) was used to culture HK-2 cells (the human proximal tubular epithelial cell line) in an incubator with 5% CO2 at 37°C. After overnight culture in serum-free medium, the HK-2 cells were pretreated with 100 μmol/L melatonin (Selleck, USA) for 1 h, followed by high glucose (30 mmol/L) intervention for 24 h. The cells were collected for subsequent experiments.

### DCFDA staining

The cells with different interventions were washed with PBS and then incubated with DCFDA (1:1000, Invitrogen) in the dark for 30 min, followed by fluorescence microscopy to assess ROS production in cells.

### Confocal

Cells in different groups were stained with MitoTracker (Invitrogen, M22426, 1:1000) for 8 min and then fixed and blocked, followed by LC3B (Proteintech, 14600-1-AP, 1:200) incubation overnight at 4°C. A fluorescence-conjugated secondary antibody was used to incubate cells for 1 h at room temperature. Then, the nucleus was stained and photographed.

### Statistical analyses

The experimental data were analyzed by SPSS 13.0 software. The results are presented as the means ± SD. The differences among the groups were compared using one-way ANOVA. Statistical significance was indicated at a P value less than 0.05.

## Results

### Melatonin ameliorated biochemical indices and pathological damage in diabetic nephropathy mice

**Figure 1:**
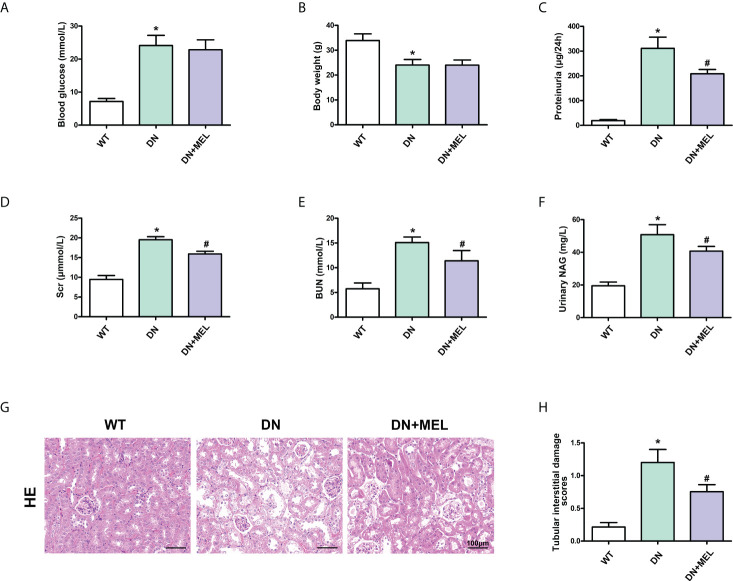
The effects of melatonin on biochemical indices and renal pathological changes in the kidneys of DN mice. The blood glucose **(A)**, body weight **(B)**, proteinuria **(C)**, serum creatinine **(D)**, BUN **(E)**, and urinary NAG levels **(F)** in different groups of mice. HE staining of renal paraffin tissue in different groups **(G)**. Tubular injury as assessed by tubular interstitial damage scores **(H)**. The values are the mean ± SD. n = 6/group. *p < 0.05 compared with the control group; ^#^p < 0.05 compared with the DN group.

Significantly increased blood glucose levels (**Figure 1A**), and reduced body weight (**Figure 1B**) were observed in the STZ-induced DN mouse model. In addition, the levels of proteinuria (**Figure 1C**), serum creatinine (**Figure 1D**), BUN (**Figure 1E**), and urinary NAG (**Figure 1F**) were notably increased in DN mice compared with in the control group, while the intervention of melatonin could mitigate these adverse changes in addition to the blood glucose level and body weight. Furthermore, HE staining showed that an increase in glomerular matrix, dilation of tubules, and exfoliation of nuclei were observed in the kidneys of DN mice compared to the control (**Figure 1G**), and this pathological renal injury was obviously relieved by melatonin treatment, as indicated by tubular interstitial damage scores (**Figure 1H**).

### Melatonin ameliorated oxidative stress and fibrosis in the kidney of DN mice

**Figure 2:**
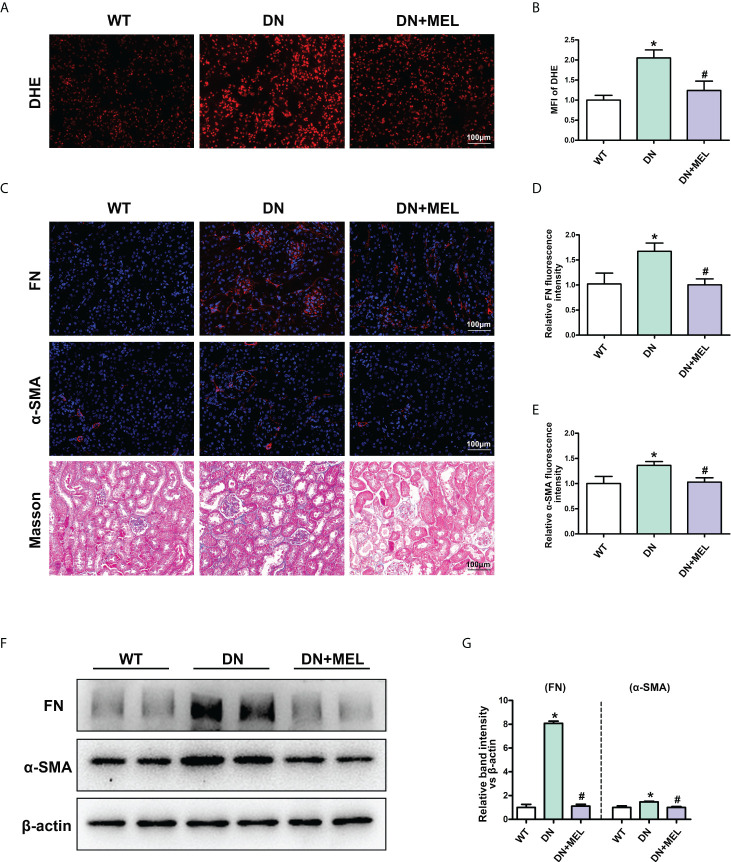
The effects of melatonin on oxidative stress and fibrosis in the kidneys of DN mice. DHE staining in the kidneys of different groups **(A, B)**. IHF analysis of FN (upper panel) **(C, D)** and α-SMA (middle panel) **(C. E)** in the kidneys of different groups. Masson staining of renal paraffin tissue in different groups (**C**, lower panel). Western blot analysis revealed the expression of FN and α-SMA **(F, G)**. The values are the mean ± SD. n = 6/group. *p < 0.05 compared with the control group; ^#^p < 0.05 compared with the DN group.

DHE staining showed that ROS levels were increased significantly in the kidneys of DN mice compared to the control, while melatonin significantly downregulated oxidative stress levels (**Figures 2A, B**). The level of renal fibrosis was determined by the expression level of fibrotic proteins (FN and α-SMA) and Masson staining. IHF staining showed increased expression of FN (**Figures 2C, D**) and α-SMA (**Figures 2C, E**) in DN mice. Masson staining also revealed increased levels of tubulointerstitial fibrosis in the kidneys of DN mice. Interestingly, these adverse changes were ameliorated by melatonin treatment (**Figures 2C–E**). To further confirm the above results, the expression levels of FN and α-SMA were detected by WB analysis. Similar results were obtained: the expression levels of FN and α-SMA were upregulated in DN mice, and melatonin downregulated their expression levels (**Figures 2F, G**).

### Melatonin promoted phosphorylation of AMPK and inhibits inflammation in the kidney of DN mice

**Figure 3:**
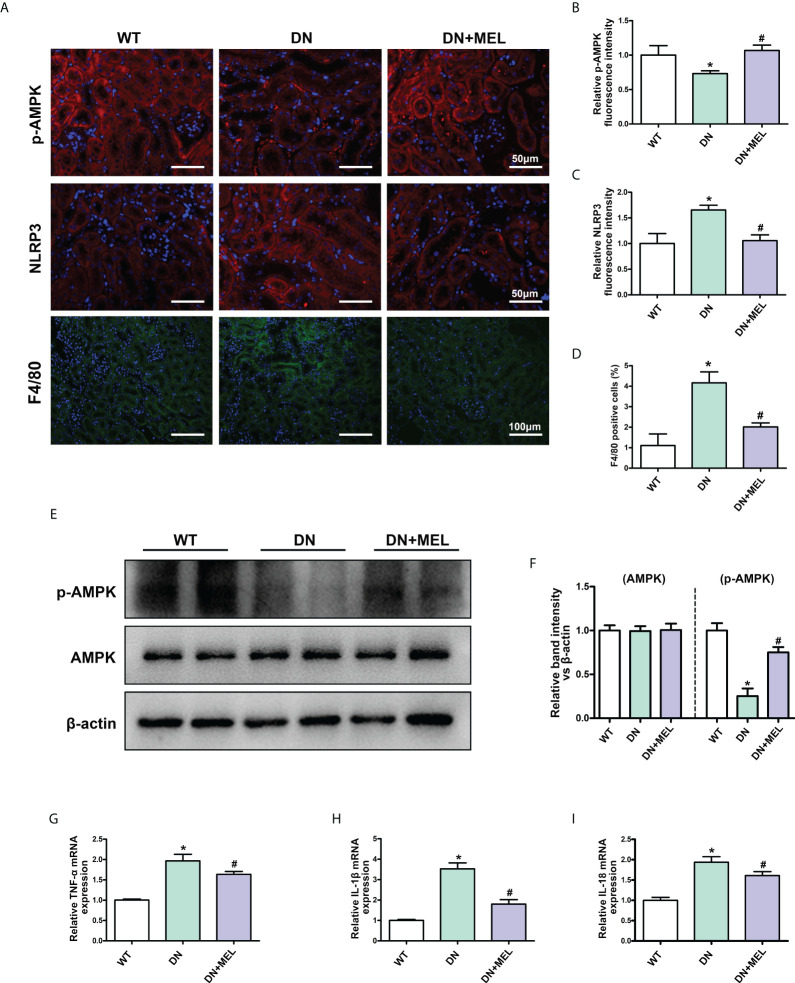
The effects of melatonin on the expression of p-AMPK and inflammation in the kidneys of DN mice. IHF analysis of p-AMPK (**A**, upper panel, **B**), NLRP3 (**A**, middle panel, **C**), and F4/80 (**A**, lower panel, **D**) in the kidneys of different groups. Western blot analysis revealed the expression of p-AMPK and AMPK **(E, F)**. The mRNA levels of TNF-α **(G)**, IL-1β **(H)**, and IL-18 **(I)** in the different groups. The values are the mean ± SD. n = 6/group. *p < 0.05 compared with the control group; ^#^p < 0.05 compared with the DN group.

IHF staining showed that the downregulated p-AMPK expression was observed in the kidneys of DN mice (**Figures 3A, B**), which was accompanied by upregulated NLRP3 expression (**Figures 3A, C**) and increased F4/80 positive cells (infiltration of macrophages) (**Figures 3A, D**). These changes were reversed by melatonin treatment. Moreover, WB analysis also showed decreased p-AMPK expression in DN mice, and melatonin reversed the downregulation of p-AMPK induced by diabetes (**Figures 3E, F**). There were no significant changes in total AMPK expression. Similarly, the mRNA levels of the inflammatory cytokines TNF-α (**Figure 3G**), IL-1β (**Figure 3H**), and IL-18 (**Figure 3I**) were increased in the kidneys of DN mice, while melatonin treatment relieved inflammation.

### Melatonin restored mitophagy dysfunction in the kidney induced by diabetes

**Figure 4:**
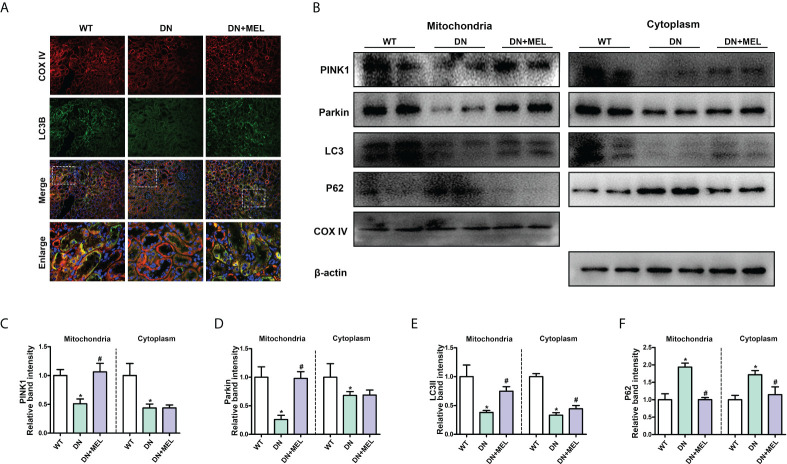
The effects of melatonin on mitophagy in the kidneys of DN mice. Costaining for COX IV (A, upper panel) and LC3B (A, second panel) observed the mitophagy changes. Western blot analysis of PINK1, Parkin, Parkin, LC3II, and P62 protein expression in mitochondria (left panels) and cytoplasm (right panels) **(B)**. Quantitative analysis of PINK1 **(C)**, Parkin **(D)**, LC3II **(E)**, and P62 **(F)**. The values are the mean ± SD. n = 6/group. *p < 0.05 compared with the control group; ^#^p < 0.05 compared with the DN group.

As shown in **Figure 4A**, mitophagy was observed by costaining mitochondrial protein (COX IV, red) and LC3B (green) in the paraffin section of kidney tissue and the overlapping regions (yellow) represented mitophagy. Compared with the control group, mitophagy activity (yellow area) was significantly reduced in the kidney of the DN group, while it was reactivated after melatonin administration (**Figure 4A**). Furthermore, to evaluate mitophagy more accurately, we separated the mitochondria and cytoplasm to observe the expression changes of crucial proteins in mitophagy. WB analysis showed that HFD+STZ treatment significantly downregulated PINK1 and Parkin expression levels in both the mitochondria and cytoplasm. Interestingly, melatonin significantly increased the expression of PINK1 and Parkin in mitochondria but did not affect their expression in the cytoplasm (**Figures 4B–D**). These results indicated that PINK1 and Parkin located in mitochondria are increased in response to melatonin. Moreover, downregulated LC3II expression and upregulated P62 expression (This represents the fluency of autophagy flux) were observed in both the mitochondria and cytoplasm of DN mice, while melatonin reversed these changes (**Figures 4B, E, F**). This finding indicates that mitophagy is inhibited in DN kidneys and that melatonin can reactivate mitophagy in renal tubular cells.

### Melatonin restored mitophagy by promoting phosphorylation of AMPK in HK-2 cells

**Figure 5:**
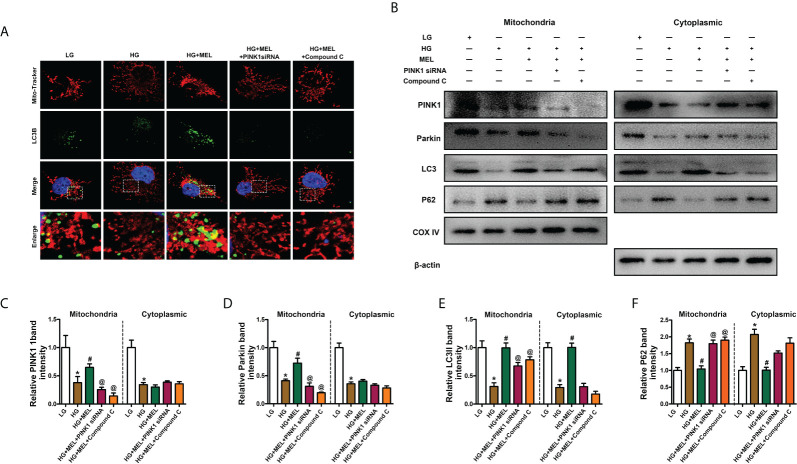
Metformin activated mitophagy in HK-2 cells treated with HG through the p-AMPK-PINK1 pathway. Mitophagy was detected by co-staining of MitoTracker and LC3B **(A)**. Western blot analysis of PINK1, Parkin, Parkin, LC3II, and P62 protein expression in mitochondria (left panels) and cytoplasm (right panels) **(B)**. Quantitative analysis of PINK1 **(C)**, Parkin **(D)**, LC3II **(E)**, and P62 **(F)**. The values are the mean ± SD. n = 4/group. *p < 0.05 compared with the control group; ^#^p < 0.05 compared with the HG group. ^@^p < 0.05 compared with the HG+MEL group.

To verify the protective mechanism of melatonin on diabetic kidney injury, we observed changes in mitophagy in HK-2 cells by inhibiting the expression of PINK1 and using the AMPK inhibitor Compound C. Mitophagy was detected by co-staining mitochondria (MitoTracker, red) and LC3B (green) as previously described (22), and the overlapping regions (yellow) represent mitophagy. Compared to the control, mitophagy was obviously inhibited in HK-2 cells treated with HG, while the effect of HG was negated by melatonin. Furthermore, the melatonin-restored mitophagy under HG was abolished by downregulation of PINK1 or inhibition of AMPK phosphorylation (**Figure 5A**). Moreover, we separated the mitochondria and cytoplasm of HK-2 cells to confirm whether the translocation of PINK1 and Parkin to mitochondria could be inhibited by AMPK inhibition. We observed that the expression levels of PINK1 and Parkin were notably decreased.

In contrast, P62 expression was increased in both the mitochondria and cytoplasm of HK-2 cells under HG conditions, and melatonin promoted the translocation of PINK1 and Parkin from the cytoplasm to mitochondria and did not significantly affect their expression levels in the cytoplasm (**Figures 5B–D**). The protective effects of melatonin on the kidney and the expression changes in LC3II and P62 were partially abolished by treatment with Pink1 siRNA or Compound C (**Figures 5B, E, F**).

### Melatonin relieved inflammation and fibrosis in HK-2 cells treated with HG

**Figure 6:**
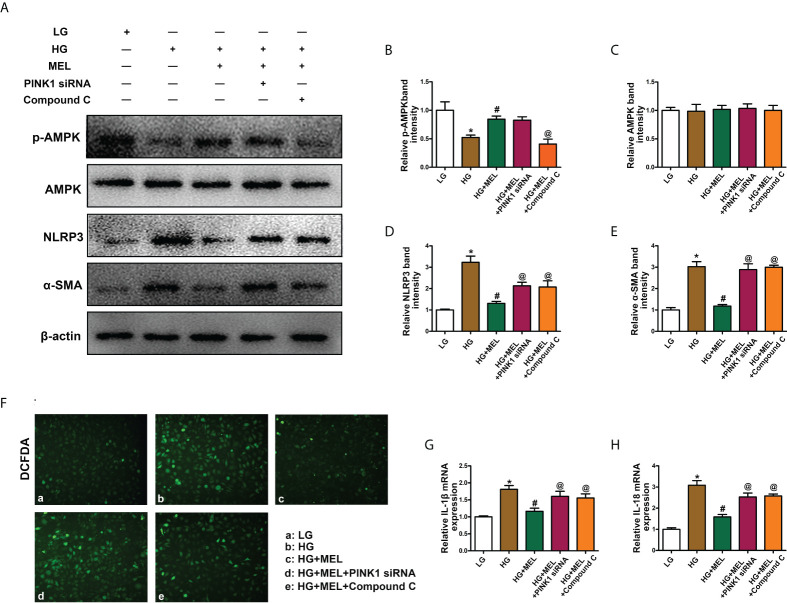
The effect of metformin on oxidative stress, inflammation, and fibrosis in HK-2 cells treated with HG. Western blot analysis of p-AMPK **(A, B)**, AMPK **(A, C)**, NLRP3 **(A, D)**, and SMA **(A, E)** expression. DCFDA staining was used to detect intracellular oxidative stress **(F)**. The mRNA levels of IL-1β **(G)** and IL-18 **(H)** in the different groups. The values are the mean ± SD. n = 4/group. *p < 0.05 compared with the control group; ^#^p < 0.05 compared with the HG group. ^@^p < 0.05 compared with the HG+MEL group.

Then, we explored the effects of melatonin on inflammation and fibrosis. WB analysis showed decreased p-AMPK expression in HK-2 cells treated with HG treatment, and melatonin restored p-AMPK expression (**Figures 6A, B**). There were no apparent changes in total AMPK expression (**Figures 6A, C**). In addition, the expression levels of NLRP3 and α-SMA were significantly increased in HK-2 cells under HG conditions, and melatonin led to the restoration of HG-induced NLRP3 and α-SMA expression. At the same time, the effect was partially inhibited by PINK1 siRNA or Compound C (**Figures 6A, D, E**). Similarly, intracellular oxidative stress levels and the mRNA levels of IL-1β and IL-18 were increased under HG conditions, while melatonin reduced the increased expression induced by HG, and PINK1 siRNA or Compound C partially blocked the effects of melatonin (**Figures 6F–H**).

## Discussion

Previous studies have considered that the renal damage caused by diabetes is mainly in the glomerulus, but the latest study shows that renal tubular injury is independent of the glomerulus and even earlier than the glomerulus (7). Multiple factors have been revealed to cause diabetes-induced renal damage, such as reactive oxygen species (23) and advanced glycosylation end products (24), which can lead to tubulointerstitial inflammation and fibrosis, promoting the progression of DN. However, these hypotheses cannot fully explain the pathogenesis of renal injury in DN. This study revealed that melatonin upregulated mitophagy by activating AMPK, alleviating renal inflammation and interstitial fibrosis.

As a highly metabolized organ, tubule cells need a large number of mitochondria to ensure their reabsorption function. Oxidative phosphorylation (OXPHOS) in mitochondria is the primary source of ATP production, and this process involves producing a large number of reactive oxygen species. Damaged mitochondria need to be removed in time; otherwise, the substances in the damaged mitochondria will leak into the cytoplasm, further aggravating cell damage. Zhan et al. demonstrated that there was a large amount of mitochondrial fragmentation accompanied by increased production of ROS and increased apoptosis in the kidneys of DN mice, while the use of drugs to restore mitochondrial function can alleviate kidney damage (25). A similar result was also observed by Ward et al. when db/db mice were treated with Mito. Q, a mitochondria-targeted protective agent, and diabetes-induced renal damage was significantly improved compared to the control (26). These evidences suggest that mitochondrial dysfunction plays a vital role in renal injury in DN. Autophagy is the process by which cells remove excess or damaged organelles in time to adapt to changes in the environment (27). Autophagy can be divided into macroautophagy (28), microautophagy (29), and chaperone-mediated autophagy (CMA) (30), according to the occurrence process. Macroautophagy can be divided into mitophagy (31), ER-phagy (32), lipophagy (33), and so on. Mitophagy can temporarily remove damaged mitochondria from cells to prevent them from releasing contents and exacerbating cell damage. Several studies have revealed that disordered mitophagy can promote the progression of DN. There were mitophagy defects, decreased mitochondrial membrane potential, and increased mitochondrial reactive oxygen species (mtROS), accompanied by downregulated PINK and Parkin expression and increased apoptosis in the kidney of db/db mice (34). A similar result was also observed by Lu et al. Mitophagy was destroyed, and apoptosis was increased in HK-2 cells treated with high glucose, while mitophagy was restored with reduced apoptosis and alleviated kidney damage (35). This study noted that the inhibition of mitophagy was accompanied by increased oxidative stress, inflammation, and apoptosis in HFD+STZ induced DN mice and HG-treated HK-2 cells. Moreover, after the isolation of mitochondria, we found that the levels of PINK1 and Parkin (the critical proteins of mitophagy) located in mitochondria were reduced, while their levels in the cytoplasm did not change significantly. This evidence suggests that there is serious mitophagy disorder in renal tubular cells in DN.

Melatonin is a pineal hormone associated with circadian rhythms, and it can also be synthesized in extra-pineal tissues such, as the heart, liver, placenta, skin, kidney, and intestine (36–38). It regulates various life activities, such as energy metabolism, lipid regulation and reproduction, pregnancy, and fetal development (39). Recently, the relationship between melatonin and autophagy has been partially revealed. Stacchiotti et al. have shown that melatonin could alleviate liver metabolism and steatosis, restore autophagy flux and ameliorate mitochondrial damage in the liver of high-fat-fed mice (40). Similarly, Wang et al. demonstrated that melatonin could regulate the interaction between autophagy and apoptosis through SIRT3, thereby alleviating cadmium-induced testicular injury (41). However, in kidney disease, the relationship between melatonin and autophagy, especially mitophagy, has rarely been studied. It has been reported that melatonin could alleviate mitochondrial oxidative damage by promoting AMPK phosphorylation (42). AMPK phosphorylation is inhibited in DN kidneys (43, 44). In addition, studies have confirmed that AMPK was a key molecule regulating mitophagy activity (45, 46). Activation of mitochondrial autophagy can ensure timely clearance of damaged mitochondria, thereby reducing oxidative stress and cellular inflammation (7). Therefore, AMPK and mitophagy may be potential targets of melatonin in DN. Here, we found that melatonin could restore the decreased mitophagy activity caused by diabetes in the kidneys of DN mice or HG-treated HK-2 cells. Mechanistically, melatonin activates mitophagy by promoting AMPK phosphorylation. The expression of PINK1 and Parkin in mitochondria was increased in the kidneys of DN+MEL mice and HG treated HK-2 cells compared to the control. At the same time, their expression did not change significantly in the cytoplasm. This suggests that melatonin can dramatically promote the activation of mitophagy. Interestingly, when PINK1 or AMPK was inhibited, the mitophagy activity restored by melatonin was also inhibited. This evidence further suggests that melatonin activates mitophagy by promoting AMPK phosphorylation. However, what is the molecular mechanism by which melatonin regulates AMPK activity? Rui et al. demonstrated that melatonin could downregulate cAMP levels through melatonin 1A receptor (MT1R), thereby activating AMPK phosphorylation to ameliorate lipid metabolism (47). Thus, the MT1R-cAMP axis is the potential mechanism by which melatonin activates AMPK in the regulation of mitophagy.

Although this study showed that melatonin played a renoprotective role in DN through the AMPK-PINK1-mitophagy pathway, there are still some problems to be solved in the future in this research. What is the molecular mechanism by which melatonin promotes AMPK phosphorylation? Can the simultaneous intervention of melatonin and Compound C (an AMPK inhibitor) in diabetic mice block the protective effect of melatonin in the kidneys of diabetic mice? Although there are some limitations, this study shows a protective effect of melatonin in DN, which is expected to be used to treat DN in the near future.

## Data availability statement

The raw data supporting the conclusions of this article will be made available by the authors, without undue reservation.

## Ethics statement

The clinical and animal study was reviewed and approved by the Medical Ethics Committee of Central South University.

## Author contributions

HT designed the study, analyzed the data, interpreted the results, and drafted the manuscript. MY, YL, XZ, SL, HL, and LS contributed to the data collection and manuscript revision. PS was the corresponding author and was involved in the study design, data interpretation, and manuscript revision. All authors contributed to the article and approved the submitted version.

## Funding

This work was supported by National Natural Science Foundation of China (81800649), Natural Science Foundation of Hunan Province (2022JJ30835,2021JJ30942,2021JJ30986), Changsha Municipal Natural Science Foundation (kq2202401, kq2014235) and General Project of Scientific Research Project of Hunan Provincial Health and Family Planning Commission (20200807).

## Conflict of interest

The authors declare that the research was conducted in the absence of any commercial or financial relationships that could be construed as a potential conflict of interest.

## Publisher’s note

All claims expressed in this article are solely those of the authors and do not necessarily represent those of their affiliated organizations, or those of the publisher, the editors and the reviewers. Any product that may be evaluated in this article, or claim that may be made by its manufacturer, is not guaranteed or endorsed by the publisher.
